# Mechanisims of asthma and allergic disease – 1082. Relevance of IL-17A to eosinopil accumulation and mucosal remodeling in chronic rhinosinusitis and nasal polyps

**DOI:** 10.1186/1939-4551-6-S1-P78

**Published:** 2013-04-23

**Authors:** Katsuhisa Ikeda, Takeshi Kusunoki, Noritsugu Ono

**Affiliations:** 1 Otorhinolaryngology, Juntendo Univerisry Faculty of Medicine, Japan

## Background

Although chronic rhinosinusitis (CRS) is a multifactorial disease in a heterogenous group of diseases, with different underlying etiologies and pathophysiologies, Japanese patients with CRS with nasal polyps are known to show two distinct phenotypes, i) tissue eosinophilia characterized by Th2-polarization (Ahmed & Ikeda, 2005) and marked expression of eotaxins (Yao, Ikeda, et al., 2009) and ii) poorly expressed eosinophilia characterized by Th1-shifted immunity and prominent expression of IL-8 (Suzuki & Ikeda, 2002).

## Methods

A new paradigm of Th17 has been applied to the recruitment of eosinophils and the remodeling of the nasal polyps of CRS (Saitoh, et al., 2010). On the other hand, IL-17 is known to restore neutrophil recruitment resulting in reduced bacterial burden in the lower airway. We found no significant difference in the bacterial features of the maxillary sinuses between eosinophilic and neutrophilic CRS with nasal polyps (Hirotsu, Ikeda, et al., 2011). Thus, underlying pathogeneses of both eosinophilic and neutrophilic polyps could be attributed to the presence of bacteria acting through different mechanisms.

## Results

The fibroblast, one of the main cell types making up nasal polyps, is actively involved in the accumulation of the extracellular matrix and is thought to be a target cell of various cytokines. Subcultured fibroblasts established from human polyp tissues expressed the IL-17A receptor. Simultaneous quantification of 27 kinds of cytokines and chemokines in culture supernatants was performed with a human multiplex cytokine assay system. There were different patterns of basal and IL-17A-mediated secretion of several cytokines and chemokines between the fibroblasts cultured from the eosinophilic and non-eosinophilic polyps. Recent progress in our laboratory found a significant correlation between IL-17A and MUC5AC positive cells, suggesting a relevance of IL-17A to the mucosal remodeling in eosinophilic-dominant pathology (Kusunoki et al., 2012). Furthermore, antioxidant systems such as Cu-Zn superoxide dismutase and heme oxygenase in the sinonasal mucosa were siginificantly suppressed in eosinophilic CRS (Kawano et al., 2012).

## Conclusions

IL-17A and its derivatives may play a central role in pathogenesis and regulation of eosinophilic dominant pathology in CRS.

